# Bespoke Biomolecular Wires for Transmembrane Electron Transfer: Spontaneous Assembly of a Functionalized Multiheme Electron Conduit

**DOI:** 10.3389/fmicb.2021.714508

**Published:** 2021-08-16

**Authors:** Samuel E. H. Piper, Marcus J. Edwards, Jessica H. van Wonderen, Carla Casadevall, Anne Martel, Lars J. C. Jeuken, Erwin Reisner, Thomas A. Clarke, Julea N. Butt

**Affiliations:** ^1^School of Chemistry and School of Biological Sciences, University of East Anglia, Norwich, United Kingdom; ^2^Yusuf Hamied Department of Chemistry, University of Cambridge, Cambridge, United Kingdom; ^3^Institut Laue-Langevin, Grenoble, France; ^4^School of Biomedical Sciences, University of Leeds, Leeds, United Kingdom

**Keywords:** cytochrome, electron transfer, microbial fuel cell, microbial electrosynthesis, photosensitizer, biomolecular wire, SANS, *Shewanella*

## Abstract

*Shewanella oneidensis* exchanges electrons between cellular metabolism and external redox partners in a process that attracts much attention for production of green electricity (microbial fuel cells) and chemicals (microbial electrosynthesis). A critical component of this pathway is the outer membrane spanning MTR complex, a biomolecular wire formed of the MtrA, MtrB, and MtrC proteins. MtrA and MtrC are decaheme cytochromes that form a chain of close-packed hemes to define an electron transfer pathway of 185 Å. MtrA is wrapped inside MtrB for solubility across the outer membrane lipid bilayer; MtrC sits outside the cell for electron exchange with external redox partners. Here, we demonstrate tight and spontaneous *in vitro* association of MtrAB with separately purified MtrC. The resulting complex is comparable with the MTR complex naturally assembled by *Shewanella* in terms of both its structure and rates of electron transfer across a lipid bilayer. Our findings reveal the potential for building bespoke electron conduits where MtrAB combines with chemically modified MtrC, in this case, labeled with a Ru-dye that enables light-triggered electron injection into the MtrC heme chain.

## Introduction

Dissimilatory metal-reducing bacteria (DMRB) are able to gain energy for growth by coupling the oxidation of organic compounds to the reduction of iron- and manganese-containing minerals. These terminal respiratory electron acceptors are insoluble. They cannot enter the bacterial cell and DMRB have evolved mechanisms to transport electrons out of the cell across otherwise electrically insulating lipid membranes (White et al., 2016; Santoro et al., 2017). The same mechanisms allow respiration on numerous extracellular electron acceptors including suitably poised electrodes. Thus, DMRB attract much attention for their abilities to deliver clean energy and chemicals (Kato, 2015; Schroder et al., 2015; Nealson, 2017; Santoro et al., 2017) in addition to their fascinating microbiology.

**FIGURE 1:**
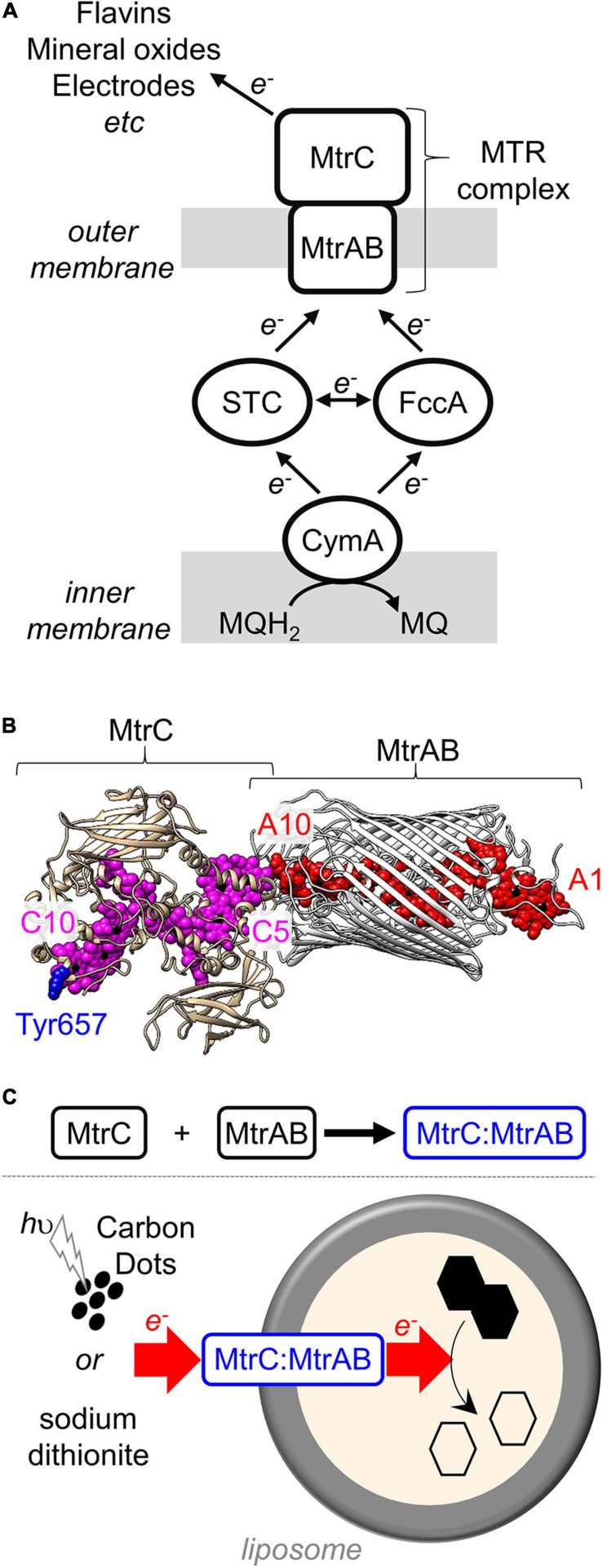
The MTR complex of *Shewanella*. **(A)** Schematic of the electron transfer pathway in *S. oneidensis* MR-1 from menaquinol (MQH_2_) to external terminal respiratory electron acceptors. **(B)** Model for the MTR complex of *S. oneidensis* MR-1 based on the crystal structure (Edwards et al., 2020) of *S. baltica* MTR complex. MtrAB (gray) showing the hemes (red). MtrC (tan) illustrating the location of the hemes (purple) and Tyr657 (blue). Hemes numbered according to their order of attachment to the primary sequence. Image rendered with Chimera. **(C)** Schematic of the *in vitro* association of MtrC and MtrAB (above) and trans-membrane electron transfer (below) through the resultant MtrC:MtrAB complex in proteoliposomes carrying the azo-dye cargo (black hexagons).

*Shewanella oneidensis* MR-1 is a model organism for fundamental and applied studies of DMRB (Fredrickson et al., 2008; Shi et al., 2012; Breuer et al., 2015; Bursac et al., 2017). The primary mechanism of electron release from *S. oneidensis* MR-1 is relatively simple (Figure 1A) and at the molecular level is arguably the best understood of the DMRB. Electrons from the oxidation of organic compounds are transferred *via* menaquinol to the inner membrane quinol dehydrogenase CymA (McMillan et al., 2012). Periplasmic cytochromes STC and FccA then transfer electrons from CymA to the outer membrane-associated MTR complex (Sturm et al., 2015). At the cell surface, electrons are transferred from the MTR complex to terminal respiratory acceptors either directly or *via* flavin mediators (von Canstein et al., 2008; Marsili et al., 2008). Alongside transmembrane electron transfer, the MTR complex is proposed to transport protons across outer membranes as the rate-limiting event (Okamoto et al., 2017) during electron transfer from biofilms of *S. oneidensis* to electrodes.

The MTR complex (Edwards et al., 2020) is composed of three proteins (Figure 1B). Two of these proteins form an outer membrane spanning complex, MtrAB, which assembles as a naturally insulated biomolecular wire with both a structure and function that are analogous to those of an electrical cable. MtrA binds an approximately linear chain of 10 *c*-type hemes which spans the lipid bilayer. Those hemes are insulated from the membrane by embedding within a 26 strand beta-barrel formed by MtrB. Electrons enter MtrAB at Heme A1 in the periplasm. At the external face of MtrAB, Heme A10 is positioned close to Heme C5 in the decaheme cytochrome MtrC (heme edge-to-heme edge distance of 8 Å) (Edwards et al., 2020). By this means, electrons can transfer from MtrA to MtrC for distribution across 10 hemes and a large surface area of MtrC that can be accessed by extracellular electron acceptors. MtrC may also pass electrons to a homologous extracellular decaheme cytochrome OmcA (Shi et al., 2006).

Previously, we reported (Lockwood et al., 2018) that *in vitro* mixing of purified MtrAB and soluble forms of MtrC results in the spontaneous formation of a stable high-affinity complex. Formation of the complex was evidenced by native PAGE and analytical ultracentrifugation with both techniques describing a complex of approximately 210 kDa that is indicative of a 1:1 association of soluble MtrC (approximately 80 kDa) and MtrAB (approximately 120 kDa). However, at that time, no information was available on the structure or electron transfer properties of the *in vitro* assembled complex. We address those gaps in knowledge in this report. Utilizing small-angle neutron scattering (SANS), the complex formed spontaneously *in vitro* is shown to have a similar structure to the MTR complex purified directly from *S. oneidensis* and revealed to be equally effective in the transfer of electrons across lipid bilayers. Our results pave the way for novel synthetic biology approaches to assemble functional MtrC:MtrAB complexes in homologous and heterologous hosts, with the potential to utilize chemically modified MtrC subunits to impart non-native functionality.

## Materials and Methods

### Protein Purification and Biochemical Analyses

Y657C MtrC, lacking the lipid attachment site of wild-type MtrC and carrying a C-terminal Strep II affinity tag, and MtrAB were purified as previously described (Edwards et al., 2018; Lockwood et al., 2018; van Wonderen et al., under review) after overexpression from pBAD202D/TOPO vectors carried by a strain of *Shewanella oneidensis* MR-1 lacking the *mtr* operon. Cys-directed labeling of Y657C MtrC with [Ru(4-bromomethyl-4′-methylbipyridine)(2,2′-bipyridine)_2_](PF_6_)_2_ (HetCat, Switzerland) was performed as described previously (van Wonderen et al., 2018, under review). The labeling efficiency was close to 100% as judged by LC-MS and UV-visible absorbance spectroscopy. The Ru-dye-labeled protein is termed Ru-MtrC (Supplementary Figure 2). For LC-MS, performed as described in van Wonderen et al. (2018), protein (typically >2 mg ml^–1^) was diluted with formic acid (0.1%, *v*/*v*) and acetonitrile (2%, *v*/*v*) prior to analysis.

Protein concentrations were quantified by absorbance spectroscopy of the fully oxidized, air-equilibrated proteins with all hemes in the Fe(III) state; for MtrAB using ε_408 nm_ = 1,238,000 M^–1^ cm^–1^ and for Ru-MtrC (van Wonderen et al., under review) using ε_408 nm_ = 1,389,000 M^–1^ cm^–1^. Extinction coefficients were experimentally determined by the pyridine hemochromagen method (Barr and Guo, 2015). Photoluminescence was measured for anaerobic protein solutions with 50 mM sodium phosphate, 50 mM NaCl, 5 mM lauryldimethylamine oxide (LDAO) (Sigma-Aldrich), pH 7.5 in sealed 1 ml quartz fluorescence cuvettes. Emission spectra were recorded using an excitation wavelength of 460 nm. Measurements were made with a Cary Eclipse Fluorescence Spectrophotometer: excitation slit width, 20 nm; emission slit width, 10 nm; and PMT detector voltage, medium.

Gel filtration chromatography was carried out at 4°C using a Superose 6 Increase 10/300 column (Cytiva) operated by an Äkta Pure chromatography system. The column was equilibrated with 50 mM sodium phosphate, 50 mM NaCl, 5 mM LDAO, pH 7.5 before loading 0.5 ml of protein sample in the same buffer. The sample was eluted at a flow rate of 0.25 ml min^–1^, eluent was monitored by optical spectroscopy at 410 nm to detect the presence of heme.

### Analytical Ultracentrifugation

Sedimentation equilibrium analytical ultracentrifugation (SE-AUC) experiments were performed using a Beckman Optima XLA-I analytical ultracentrifuge equipped with scanning absorbance optics. Measurements were performed in 50 mM sodium phosphate, 50 mM NaCl, 0.1% (*v*/*v*) Triton X-100 (Acros Organics), pH 7.5 for which the density (ρ) was calculated as 1.007 g ml^–1^ using utility software in Ultrascan II (Demeler, 2005). Ultrascan II was also used to calculate the partial specific volume ($\bar{\upsilon }$) of each protein: 0.721 ml g^−1^ for Ru-MtrC, 0.716 ml g^−1^ for MtrAB, and 0.718 ml g^−1^ for the Ru-MtrC:MtrAB complex. SE-AUC was performed at 20°C using speeds of 8,000, 10,000, and 12,000 rpm with absorbance profiles recorded at 410 nm. The program Ultrascan II was used to analyze the sedimentation equilibrium profiles and to fit the data to those predicted for single non-interacting species. Data are presented as $R^{2}-R_{\text{ref}}^{2}$ against Ln(A_410 nm_); the gradient of this plot can be used to determine the molecular weight (*M*_*W*_) of the species by the equation:

$$M_{\text{W}}=\frac{2\text{R}T}{\left(1-\bar{\upsilon }\rho \right)\omega^{2}}\times \frac{d\text{ln}\left(C_{\text{r}}\right)}{d\left(R^{2}-R_{\text{ref}}^{2}\right)}$$

where ***R*** is the gas constant, *T* is the temperature, *C*_r_ is the sample concentration at radial distance *R*, *R*_ref_ is the radial distance of the sample meniscus, and *ω* is the angular velocity. Radial distance is measured from the axis of rotation.

### SANS Data Collection and Analysis

Ru-MtrC:MtrAB was prepared by combining Ru-MtrC with a slight excess of MtrAB and performing gel filtration chromatography as described above. Ru-MtrC:MtrAB eluting from the Superose 6 Increase 10/300 column with *V*_*e*_ < 16.5 ml, i.e., separated from free MtrAB, was pooled and loaded onto a 5-ml HiTrap Q FF column (Cytiva) equilibrated with 50 mM sodium phosphate, 50 mM NaCl, 5 mM LDAO, pH 7.5. Bound protein was washed with 50 ml of 20 mM HEPES, 100 mM NaCl, 2.8 mM Fos-choline 12 (Anatrace), pH 7.8 at 1 ml min^–1^ to exchange the detergent before elution with 20 mM HEPES, 0.5 M NaCl, 2.8 mM Fos-choline 12, pH 7.8. A 100-kDa molecular weight cutoff spin concentrator (Millipore) was then used to lower the NaCl concentration to 100 mM and concentrate the protein to 10 mg ml^–1^. Immediately prior to SANS data collection, the protein sample was dialyzed overnight in a sealed DURAN bottle containing 20 mM HEPES, 100 mM NaCl, 2.8 mM Fos-choline 12, 13% D_2_O, pH 7.8 using a 50-kDa molecular weight cut-off Dispo-Biodialyzer (Merck). Inclusion of 13% D_2_O ensured that the neutron scattering length density of the buffer matched that of Fos-choline 12 micelles. This match point was previously determined by a SANS study of MtrAB and the MTR complex (Edwards et al., 2018). Protein samples at concentrations of 6.3 and 3.1 mg ml^–1^ were prepared by appropriate dilution using dialysis buffer to ensure precise buffer matching. Samples of 200 microliters were centrifuged at 13,000 × *g* for 10 min at 4°C to remove any potentially aggregated material, although no visible pellet was observed, before being sealed in 0.1 cm path-length suprasil quartz cuvettes (Hellma). An aliquot of dialysis buffer was also prepared in the same manner and was measured alongside the protein sample for downstream buffer subtraction.

SANS data were collected on the D22 diffractometer (Institut Laue-Langevin, France) using a neutron beam (*λ* = 6 Å ± 10%) at three configurations of collimation, 17.6, 8.0, and 2.8 m, and respective detector distances of 17.6, 8.0, and 1.4 m covering Q ranging from 0.003 to 0.6 Å^–1^. The collimation cross-section was 40 × 55 mm, and the sample aperture was 7 × 10 mm. Exposure times ranged from 60 s to 2 h depending on sample concentration, contrast, and instrument configuration. Data reduction was performed using GRASP including blocked beam and empty cell background subtraction, sample thickness and transmission scaling, and calibration to absolute intensity using incident neutron flux at sample position. As the final step azimuthal averaging was performed to output the scattering intensity I(Q).

Data were processed as previously described (Edwards et al., 2018) for the MTR complex. Briefly, curves were merged and buffer subtraction was carried out utilizing IGOR Pro (Wavemetrics) with the NCNR macros installed (Kline, 2006). The ATSAS software suite (Manalastas-Cantos et al., 2021) was used to analyze the I(Q) curves in order to perform a Guinier analysis to estimate the radius of gyration (*R*_g_) at low *Q* (*Q*_max_ × *R*_g_ < 1.3) and to produce Kratky plots to evaluate the overall compactness of the protein complexes. GNOM (Svergun, 1992) was used to calculate the pair distance distribution function [*P*(*r*)], i.e., the Fourier inversion of the scattering intensity [*I*(*Q*)], which provides an independent estimation of *R*_g_ as well as the maximum dimension of the scattering particle *D*_max_. Using DAMMIF (Franke and Svergun, 2009), 20 bead models of the molecule were refined to fit the experimental *P*(*r*). The DAMAVER suite of software (Volkov and Svergun, 2003) was then used to create pairwise alignments of all 20 models, identify and remove outliers among the models, 2 of the 20 models in this case, and create an average from the remaining 18 models. Reported molecular envelopes were produced by refinement of the averaged model using DAMMIN (Svergun, 1999). A homology model for the MTR complex of *S. oneidensis* (Figure 1B) was prepared by using Phyre2 to generate a model for MtrAB and combining that with the crystal structure of *S. oneidensis* MtrC (PDB ID: 4LM8) docked in the same position as MtrC of the *S. baltica* MTR complex (PDB ID: 6R2Q). This homology model was aligned to the SANS molecular envelopes using SUPCOMB (Kozin and Svergun, 2001). SANS data collection and processing statistics are reported in Supplementary Table 3.

### Photoreduction of Ru-MtrC and Ru-MtrC:MtrAB Suspensions

Experiments were performed in anaerobic 50 mM Tris:HCl, 10 mM KCl, 100 mM EDTA, and 0.2% (*v*/*v*) Triton X-100, pH 8.5. Spectra were recorded in 1 ml SOG cuvettes (Hellma) in a Biochrom WPA Biowave II Diode-array spectrophotometer placed in a N_2_-filled chamber (Belle Technology, atmospheric O_2_ < 5 ppm). An Omega Optical 475RB Notch filter was used to prevent photoexcitation by the spectrophotometer. The light source for photoreduction was a Thorlabs mounted LED (*λ*_max_ = 450 nm) (Supplementary Figure 1) equipped with a collimator adapter. The excitation intensity at the sample was 110 W m^–2^ (0.42 mE m^–2^ s^–1^) as determined by potassium ferrioxalate actinometry (Hatchard and Parker, 1956; Pitre et al., 2015). Samples were irradiated continuously from above and spectra taken at the desired time intervals. The percentage of reduced hemes was quantified using the baseline-corrected absorbance of the heme Soret band at 420 nm. The absorbance prior to irradiation was taken to be of the fully oxidized protein (0% reduced heme). The absorbance of fully reduced protein (100% reduced heme) was obtained at the end of the experiment by addition of an excess of the chemical reductant sodium dithionite.

### Preparation of Liposomes and Proteoliposomes

Liposomes were prepared following an adaptation of the method reported by Stikane et al. (2019). Twenty milligrams of Polar lipid extract (Avanti Polar Lipids) was suspended in 750 μl of 50 mM Tris:HCl, 10 mM KCl, and 10 mM reactive red 120 (RR120, Sigma-Aldrich), pH 8.5 by vigorous vortexing for 20 min. The suspended lipid was then solubilized by addition of 500 μl of 250 mM octyl glucoside (Anatrace). Proteins were then incorporated by addition of 100 μl of 25 μM protein complex in 50 mM sodium phosphate, 50 mM NaCl, and 5 mM LDAO, pH 7.5. Ru-MtrC and MtrAB were mixed to form the Ru-MtrC:MtrAB complex prior to incorporation of this complex into proteoliposomes. For control experiments without proteins, liposomes were prepared by addition (100 μl) of 50 mM sodium phosphate, 50 mM NaCl, and 5 mM LDAO, pH 7.5. The resulting mixture was incubated on ice for 20 min before gradual addition, over 2 min, to ice-cold 50 mM Tris:HCl, 10 mM KCl, and 10 mM RR120, pH 8.5 to give a final volume of 50 ml. This dilution lowered the octyl glucoside concentration below its critical micelle concentration resulting in liposome formation with spontaneous protein integration and RR120 encapsulation.

The dilute liposome suspension was subjected to ultracentrifugation at 205,000 × *g* for 1 h to pellet the liposomes. After removal of the supernatant, pellets were transferred to a N_2_-filled chamber and resuspended in 50 ml of anaerobic 50 mM Tris:HCl and 10 mM KCl, pH 8.5. The liposome pellets recovered after a second ultracentrifugation, as above, were then resuspended to a final volume of 1 ml in anaerobic 50 mM Tris:HCl and 10 mM KCl, pH 8.5 and allowed to equilibrate overnight at room temperature in the anaerobic chamber. The liposome concentration of these samples was approximately 300 nM, estimated on the basis of the liposome dimensions (see below) and lipid composition as described in Supplementary Material.

Dynamic light scattering was used to assess the dimensions of the liposomes in samples containing approximately 6 nM liposome in 50 mM Tris:HCl and 10 mM KCl, pH 8.5. Measurements used a Zetasizer Nano with DTS1070-folded capillary cells (Malvern Panalytical, Malvern, United Kingdom), and zeta potentials were measured using the same equipment. Samples were equilibrated at 25°C for 2 min prior to measurement, and the solvent viscosity was considered to be that of water. The identity of proteins in each of the samples was confirmed using SDS-PAGE with proteins visualized by heme (Thomas et al., 1976) or Coomassie stain.

### Transmembrane Electron Transfer in (Proteo-) Liposomes

Bleaching of encapsulated RR120 was used to monitor transmembrane electron transfer with (proteo-) liposomes as illustrated schematically in Figure 1C. All experiments were performed inside a N_2_-filled chamber (Belle Technology, Weymouth, United Kingdom; atmospheric O_2_ < 5 ppm). For experiments with sodium dithionite as the electron donor, a stock solution (20 mg ml^–1^) was prepared by dissolving the required mass in anaerobic 50 mM Tris:HCl and 10 mM KCl, pH 8.5. Sodium dithionite was then added (approximately 0.1 mM final concentration) to anaerobic suspensions of (proteo-) liposomes (approximately 6 nM) containing RR120 in anaerobic 50 mM Tris:HCl and 10 mM KCl, pH 8.5 in 1 ml SOG cuvettes. Absorbance spectra were measured over 30 min. Finally, Triton X-100 was added to 0.2% (*v*/*v*) to lyse the liposomes. In all cases, this final step led to rapid bleaching of all RR120 present and demonstrated the presence of excess reductant.

Photochemically driven transmembrane electron transfer was monitored by a similar method using graphitic N-doped carbon dots as described in Martindale et al. (2017) and Supplementary Figure 3. Prior to use, the graphitic N-doped carbon dots were suspended to 1 mg ml^–1^ in anaerobic 50 mM Tris:HCl and 10 mM KCl, pH 8.5. For photoreduction, anaerobic samples in 1 ml SOG cuvettes contained approximately 6 nM (proteo-) liposomes with encapsulated RR120, 10 μg ml^–1^ of graphitic-N-doped carbon dots, and 25 mM EDTA in 50 mM Tris:HCl and 10 mM KCl, pH 8.5. Samples were irradiated by visible-light (*λ* > 400 nm) from the side using a Krüss cold light source (Supplementary Figure 1) with a fiber optic light pipe as described in Rowe et al. (2017) Light intensity was measured at 2.5 kW m^–2^ using an Amprobe Solar-100 solar power meter.

Absorbance spectra were measured at desired times with a Biochrom WPA Biowave II diode array spectrophotometer. Scattering due to the liposomes was calculated using the equation:

$$Scatteringintensity=A+B/\lambda^{4}$$

and subtracted from the measured data. For each series of experiments, the variables A and B were adjusted to give a good fit to the measured data where absorbance from protein and dye were minimal, below 260 and above 640 nm (e.g., Supplementary Figure 4). Dye absorbance was then quantified at 539 nm, a wavelength which is isosbestic with respect to heme oxidation state (Stikane et al., 2019).

## Results and Discussion

MtrAB when purified and resuspended in detergent micelles was previously (Lockwood et al., 2018) shown to bind to separately purified soluble forms of MtrC but not OmcA, which is an extracellular decaheme cytochrome of *S. oneidensis* homologous to MtrC. This selective binding led us to anticipate that the MtrC:MtrAB complex would have a structure similar to that of the MTR complex. MtrC Heme C5 would be positioned close to MtrAB Heme A10 (Figure 1B), and the environment of MtrC Heme C10 would be the same in purified MtrC as in the MTR complex. Labeling the surface of MtrC near Heme C10 with a luminescent dye would provide the opportunity to report on that local environment. If, in addition, that dye could transfer photo-energized electrons to the MtrC hemes, there might be opportunities to probe MtrC to MtrAB electron transfer within the MtrC:MtrAB complex following light-triggered electron injection into MtrC Heme C10.

The thiol-reactive dye [Ru(4-bromomethyl-4′-methylbipyridine)(2,2′-bipyridine)_2_](PF_6_)_2_ (Geren et al., 1991; Millett and Durham, 2002) has been successfully attached to cysteine residues on the surfaces of a number of redox proteins. This dye has a well-characterized luminescence that is sensitive to local environment (van Wonderen et al., 2018). Furthermore, the photoexcited dye is capable of injecting photo-energized electrons into multiheme cytochromes including *S. oneidensis* STC (van Wonderen et al., 2019) and of PpcA of *Geobacter sulfurreducens* (Kokhan et al., 2015). Thus, we prepared (van Wonderen et al., under review) a soluble MtrC variant with Tyr657 replaced by Cys on the surface of MtrC at a site close to Heme C10 (Figure 1B). Cys657 was then labeled by reaction with [Ru(4-bromomethyl-4′-methylbipyridine)(2,2′-bipyridine)_2_](PF_6_)_2_ to form a protein, here termed Ru-MtrC, that retains the spectral and redox properties of the hemes in the native protein (van Wonderen et al., under review).

As described below, Ru-MtrC forms a 1:1 complex when mixed with MtrAB. The structure of the resultant complex, termed Ru-MtrC:MtrAB, was assessed by analytical ultracentrifugation, gel-filtration chromatography, SANS, and luminescence spectroscopy. Electron transfer was probed by optical spectroscopy of the complex in detergent micelles and incorporated in lipid bilayers.

### Oligomeric State and Solution Structure of the Ru-MtrC:MtrAB Complex

**FIGURE 2:**
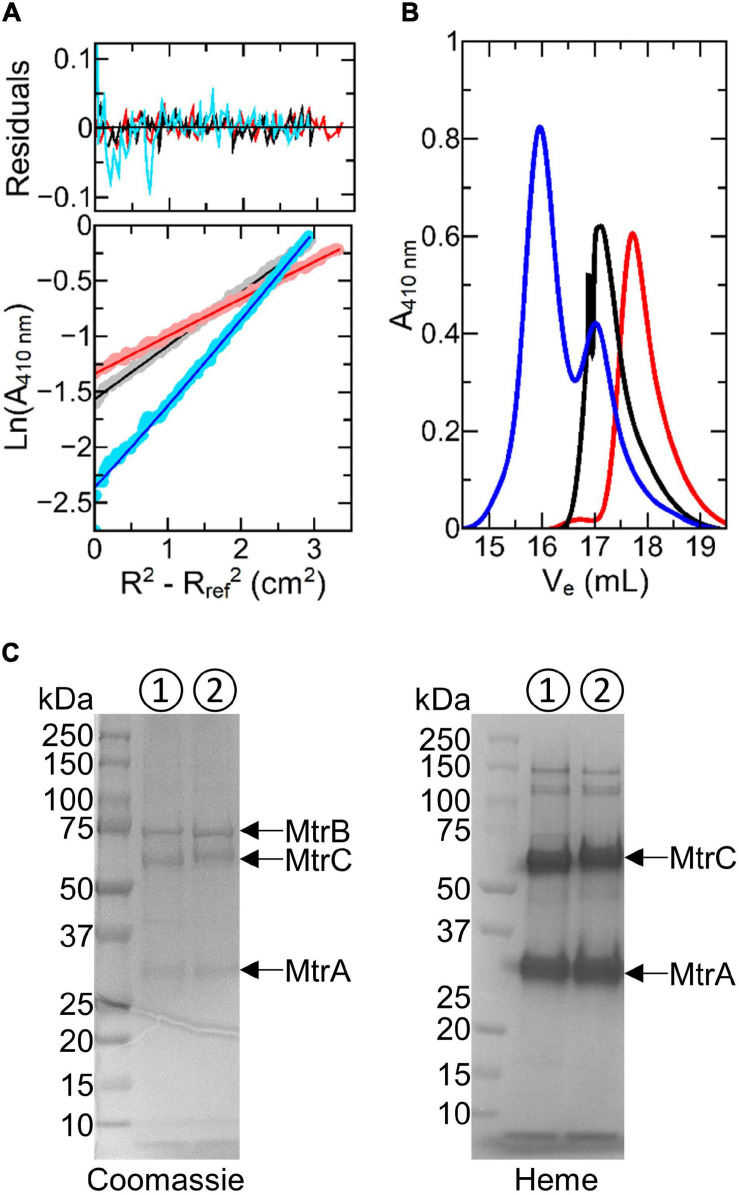
Biophysical characterization of Ru-MtrC:MtrAB. **(A)** Sedimentation equilibrium analytical ultracentrifugation traces for 0.4 μM MtrAB (black), 0.4 μM Ru-MtrC (red), and a mixture of 0.25 μM Ru-MtrC equilibrated with 0.25 μM MtrAB (blue). Samples were centrifuged at 8,000 rpm for 20 h at 20°C in 50 mM sodium phosphate, 50 mM NaCl, and 0.1% (*v*/*v*) Triton X-100, pH 7.5 to reach equilibrium. Lower panel: data (circles) and fits (lines) to the behavior for a single non-interacting species; see text for details. Upper panel: residuals. **(B)** Gel filtration chromatograms for samples of 40 μM MtrAB (black), 40 μM Ru-MtrC (red), and a mixture of 32 μM Ru-MtrC equilibrated with 48 μM MtrAB (blue). A Superose 6 Increase 10/300 column was equilibrated in 50 mM sodium phosphate, 50 mM NaCl, and 5 mM LDAO, pH 7.5. Elution was at 0.25 ml min^− 1^. **(C)** SDS-PAGE analysis of the MTR complex (➀) and Ru-MtrC:MtrAB (➁) (*V*_*e*_ < 16.5 ml from gel filtration shown in **(B)**). Proteins visualized by Coomassie and Heme stain as indicated.

SE-AUC provides a direct measure of the average mass of proteins in solution (Lebowitz et al., 2002). As a consequence, their oligomeric state can be readily defined and SE-AUC was the method of choice for initial characterization of samples containing Ru-MtrC, MtrAB, and 1:1 mixtures of Ru-MtrC with MtrAB. Data were collected at three rotation speeds for each sample. In each case, the absorbance profile indicative of protein concentration across the sample, e.g., Figure 2A, was well-described by the behavior predicted for a single, non-interacting species. For Ru-MtrC (0.4 μM), the apparent mass was 82,400 Da, and for MtrAB (0.4 μM), it was 120,000 Da. These values are in good agreement with those of 76,788 and 114,047 Da calculated for Ru-MtrC and MtrAB, respectively, on the basis of primary sequence, covalent modification by 10 *c*-type hemes, and labeling of the former protein with the Ru-dye. It was concluded that Ru-MtrC and MtrAB are monomer and heterodimer, respectively, under the experimental conditions.

Analysis of the absorbance profiles for samples containing MtrAB (0.25 μM) and an equal concentration of Ru-MtrC revealed a single homogeneous species with an apparent molecular mass of approximately 204,000 Da. This mass is comparable with the sum (190,835 Da) of those for Ru-MtrC and MtrAB. Thus, Ru-MtrC combines with MtrAB to form a heterotrimer having a 1:1 ratio of Ru-MtrC and MtrAB. We note that all samples contained 0.1% (*v*/*v*) of the detergent Triton X-100, to maintain solubility of the membrane proteins, and that the approximate micellar weight for Triton X-100 is 80 kDa. However, there is negligible micellar contribution to the overall mass for proteins of the size studied here. This is because the partial specific volume of Triton X-100 (0.91 ml g^–1^) (Paradies, 1980) is close to that of the buffer-electrolyte (0.99 ml g^–1^) such that the micelle is only weakly affected by the centrifugal force. The proteins, by comparison, have partial specific volumes of approximately 0.72 ml g^–1^.

Further evidence for spontaneous formation of a tight, stable complex between Ru-MtrC and MtrAB was provided by analytical gel filtration chromatography (Figure 2B). Resolution was afforded by a Superose 6 Increase column for samples having a concentration approximately 100× greater than used for SE-AUC analysis. Ru-MtrC eluted as a single peak centered on an elution volume (*V*_e_) of approximately 17.8 ml and samples of the higher mass MtrAB complex eluted as a single peak centered on *V*_e_ approximately 17.1 ml. A sample containing Ru-MtrC (32 μM) equilibrated with an excess of MtrAB (48 μM) eluted as two peaks. The smaller peak, centered on *V*_e_ approximately 17.1 ml, is assigned to excess MtrAB. The larger peak, centered on *V*_e_ approximately 15.5 ml, is assigned to a species of higher molecular mass that we consider to be the Ru-MtrC:MtrAB complex. This interpretation was supported by SDS-PAGE (Figure 2C) of material with *V*_e_ approximately 15.5 ml where bands with the expected mass of Ru-MtrC, MtrA, and MtrB were observed.

Previously (Edwards et al., 2018), we used SANS to resolve the molecular envelopes of MtrAB and the MTR complex purified from *S. oneidensis*. For those experiments, proteins were suspended in a buffer containing Fos-choline 12 detergent and 13% D_2_O in order to match the neutron scattering length density of both detergent micelles and bulk buffer solution. Upon buffer subtraction, the scattering intensity profile of the detergent micelles is also subtracted revealing the neutron scattering intensity profile from the protein complexes alone. We used the same approach here to resolve the molecular envelope of Ru-MtrC:MtrAB. We note that our ability to prepare Ru-MtrC:MtrAB in Triton X-100 (SE-AUC), LDAO (gel filtration), and Fos-choline 12 (SANS) highlights the stability of the complex in a range of detergents.

**FIGURE 3:**
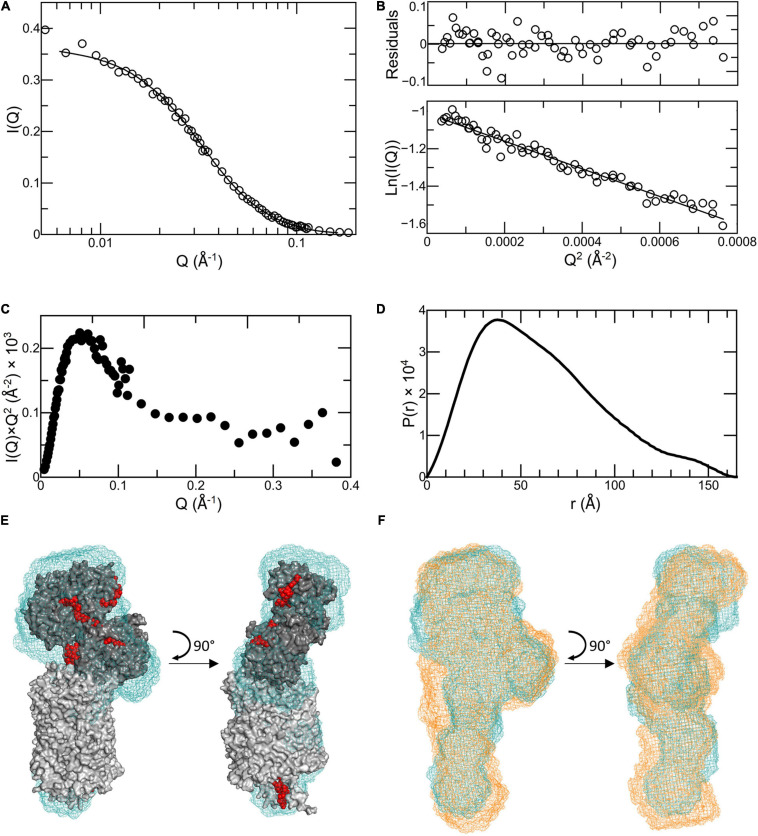
Analysis of SANS data for Ru-MtrC:MtrAB. **(A)** Scattering data (truncated to *Q* = 0.161 Å^−1^) shown as circles with the fit to the *P*(*r*) curve generated by GNOM shown as a line. **(B)** Guinier region of scattering curve, lower panel shows data as circles and a linear fit as a line. Upper panel shows residuals from fitting. **(C)** Kratky plot. **(D)** *P*(*r*) distance distribution curve. **(E)** Molecular envelope of Ru-MtrC:MtrAB (blue mesh) generated by DAMMIN aligned with the homology model of the MTR complex from *S. oneidensis* (gray with red hemes). **(F)** Molecular envelope of Ru-MtrC:MtrAB (blue mesh) aligned with that of the MTR complex (orange mesh). Source data for latter as reported in Edwards et al. (2018), see text for details.

Neutron scattering data were collected for Ru-MtrC:MtrAB at concentrations of 6.3 and 3.1 mg ml^–1^. The scattering intensity profiles scaled linearly with protein concentration indicating an absence of concentration-dependent interparticle interactions (Supplementary Figure 5). The scattering profiles were merged, providing a curve (Figure 3A) with high reliability and signal to noise. Guinier analysis (Figure 3B) produced a linear plot indicating that the samples of Ru-MtrC:MtrAB were not aggregated and had an approximate radius of gyration (*R*_g_) of 46.9 ± 0.6 Å. A Kratky plot [*I*(*Q*) × *Q*^2^ vs. *Q*, where *I*(*Q*) is the intensity at a given scattering distance and *Q* is momentum transfer] (Figure 3C) indicated Ru-MtrC:MtrAB was globular. *P*(*r*) distance distribution curves were generated using the GNOM program based on inverse Fourier transform of the data to a maximum *Q*-value of 0.161 Å^–1^. The scattering intensity decreased significantly beyond this value, so data were truncated before calculation of *P*(*r*) distribution curves. The *P*(*r*) curve shape (Figure 3D) was suggestive of a globular protein with a maximum distance in the molecule, *D*_max_, of 166 Å and an *R*_g_ of 48.2 Å. The latter is in good agreement with the value determined by Guinier analysis (see above). The theoretical scattering produced by this *P*(*r*) curve fits well to the experimental scattering data (Figure 3A) with aχ^2^ of 0.764 as determined by GNOM.

The *P*(*r*) curve was used to produce *ab initio* structural models for Ru-MtrC:MtrAB using DAMMIF (Franke and Svergun, 2009); these were then processed with DAMAVER (Volkov and Svergun, 2003) as previously described (Edwards et al., 2018). Final refinement using DAMMIN (Svergun, 1999) produced a molecular envelope which is shown in Figure 3E. It has been aligned, using SUPCOMB (Kozin and Svergun, 2001) to the homology model for the *S. oneidensis* MTR complex (Figure 1B) generated based upon the crystal structure of the MTR complex from *S. baltica* OS185 (Edwards et al., 2020) and the structure of MtrC from *S. oneidensis* MR-1 (Edwards et al., 2015). The theoretical scattering profile produced by this molecular envelope had aχ^2^-value of 2.748 (determined by DAMMIN) against the original scattering curve (Supplementary Figure 6F), indicating a good fit to the data, and the alignment had a normalized spatial discrepancy (NSD) of 2.05. The data reveal agreement between the Ru-MtrC:MtrAB molecular envelope from SANS and the homology model for the MTR complex at the level of resolution afforded by SANS.

The scattering data obtained previously (Edwards et al., 2018) for the MTR complex was subject to the same analysis and modeling comparable with that described above for Ru-MtrC:MtrAB (Supplementary Figure 6). The final molecular envelope for the MTR complex had a χ^2^-value of 1.972 (determined by DAMMIN) against the scattering data (Supplementary Figure 6F) and alignment to the homology model for *S. oneidensis* MTR complex gave a NSD of 2.72 (Supplementary Figure 6E). The DAMMIN models of the Ru-MtrC:MtrAB and MTR complexes are compared in Figure 3F and reveal very similar global structures at the resolution provided by SANS. Both models align similarly well with the homology model for the MTR complex generated from the crystal structure (Edwards et al., 2020) of the complex from *S. baltica*. This finding gives confidence in the homology model generated to describe the structure of the *S. oneidensis* MTR complex. In addition, it reveals that the MTR solution structure is not significantly different to that resolved in a crystalline state.

SANS analysis of Ru-MtrC:MtrAB lacks the resolution required to locate the Ru-dye attached to MtrC Cys657. To confirm the dye is positioned away from the interface between MtrC and MtrAB, as expected from the structure of the MTR complex (Figure 1B), the spectral properties of the dye attached to MtrC were investigated in the absence and presence of MtrAB. If MtrC Heme C10 is located near the interface with MtrAB, we can expect this to impact the photoluminescence intensity or spectral profile of the Ru-dye. However, there was negligible change to the emission spectrum of 1 μM Ru-MtrC after incubation with 0.5, 1, or 2 μM MtrAB over a 5-h period (Supplementary Figure 7). These observations are consistent with a “correct” relative orientation of Ru-MtrC and MtrAB such that Heme C10 is positioned some distance from MtrAB (Figure 1B).

### Electron Transfer Through the Ru-MtrC:MtrAB Complex

**FIGURE 4:**
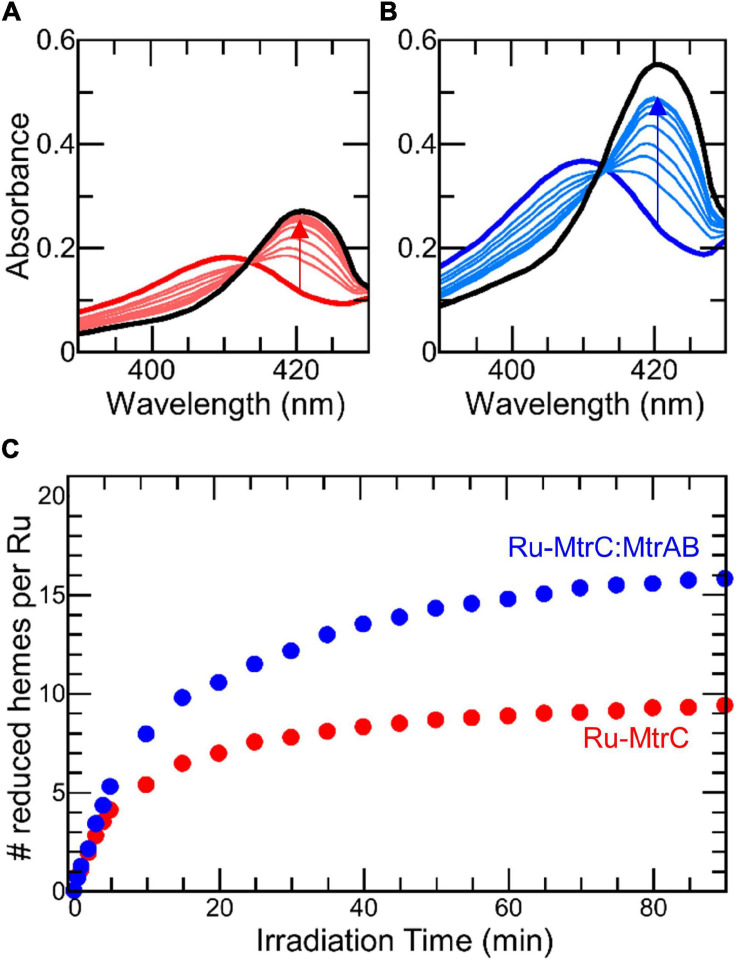
Photoreduction of Ru-MtrC and Ru-MtrC:MtrAB Suspensions. Spectra of oxidized protein (thick colored line), over 90 min irradiation (thin colored lines), and after addition of dithionite (black line) for **(A)** Ru-MtrC and **(B)** Ru-MtrC:MtrAB. Samples of Ru-MtrC (0.12 μM) and Ru-MtrC:MtrAB (0.14 μM) in anaerobic 50 mM Tris, 10 mM KCl, 100 mM EDTA, and 0.2% (*v*/*v*) Triton X-100, pH 8.5. Irradiation at 450 nm, intensity 110 W m^− 2^. **(C)** Time course of photoreduction for Ru-MtrC (red) and Ru-MtrC:MtrAB (blue). Number of reduced hemes defined by ΔA_420 nm_; see text for details.

SANS analysis suggests Ru-MtrC:MtrAB assembles in either the same or a highly similar manner to the wild-type MTR complex assembled in the outer membrane of *Shewanella*. To further investigate the *in vitro*-assembled Ru-MtrC:MtrAB complex, a series of experiments explored its electron transfer properties. Initial investigations took advantage of the Ru-dye attached to MtrC. The photoexcited triplet state of this dye, generated by absorption of photons at blue wavelengths, is a strong reductant (*E*_m_ approximately -830 mV vs. SHE) with a lifetime of approximately 600 ns that is capable of transferring its photoenergized electron to a nearby protein cofactor (e.g., Geren et al., 1991; Millett and Durham, 2002; van Wonderen et al., 2018, 2019, under review). The photoenergized electron becomes trapped in the protein if the oxidized dye is reduced by a sacrificial electron donor and provided there are no sacrificial acceptors present. For cytochromes with multiple hemes, such photoreduction is cumulative (van Wonderen et al., 2018) and readily quantified by changes in absorbance of the Soret band as illustrated for Ru-MtrC (e.g., Figure 4A). Initially, the protein is fully oxidized with all hemes in the Fe(III) state as indicated by the Soret band with maximum absorbance at 410 nm (Figure 4A, thick red line). Upon irradiation (*λ* = 450 nm) in the presence of EDTA as sacrificial electron donor, the Soret band was red shifted and gained intensity (Figure 4A, thin red lines). These changes revealed the formation of reduced Fe(II)-containing hemes. Fully reduced Ru-MtrC with 10 Fe(II) hemes produced on equilibration with an excess of the chemical reductant sodium dithionite has an intense Soret band with maximum absorbance at 420 nm (Figure 4A, black line).

**SCHEME 1:**
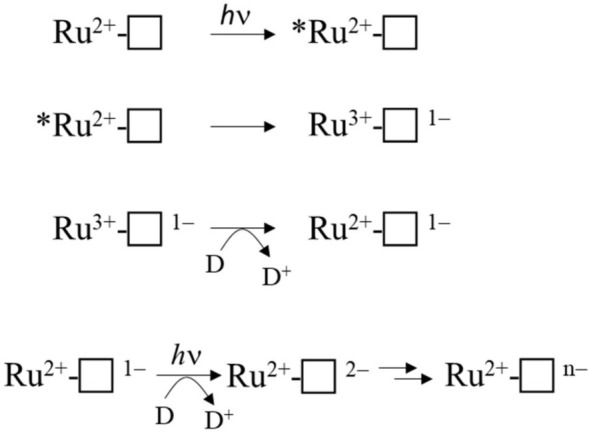
Proposed mechanism of cumulative photoreduction showing only productive steps.

Cumulative photoreduction of Ru-MtrC:MtrAB was observed during equivalent experiments (e.g., Figure 4B). Of significance was the finding that for this complex, approximately 20 min irradiation was sufficient to drive the reduction of > 10 hemes per molecule of Ru-dye (Figure 4C). It can be concluded that Ru-MtrC transfers electrons to MtrAB. Reduction was not detected in the absence of EDTA, without irradiation, and without the Ru-dye allowing us to propose a likely mechanism for the cumulative photoreduction (Scheme 1). In Scheme 1, the excited state Ru-dye is presented as ^∗^Ru^2+^, the box represents MtrC (or MtrC:MtrAB), D is the sacrificial electron donor EDTA, and only productive steps for cumulative photoreduction are illustrated.

A striking feature of the time courses for photoreduction of MtrC and MtrC:MtrAB is that the maximum (i.e., initial) rates are of the order of 1 heme min^–1^ despite predicted (Jiang et al., 2019, 2020) and measured (van Wonderen et al., under review) heme-to-heme electron transfer rates > μs^–1^. Similar behavior was described (van Wonderen et al., 2019) for the tetraheme cytochrome STC of *S. oneidensis* labeled in a manner comparable with that for Ru-MtrC described here. This situation points toward rate-limiting events that involve the Ru-dye, e.g., photoexcitation, EDTA oxidation, and/or charge separation (recombination). However, further investigation of these possibilities falls beyond the scope of this present study.

The timescale of cumulative photoreduction reported in this study makes it difficult to confidently distinguish the contribution of electron transfer within Ru-MtrC:MtrAB from that due to electron transfer between such complexes. To overcome this difficulty and provide a description of electron transfer through Ru-MtrC:MtrAB, we incorporated the complex into liposome bilayers. The proteoliposomes, illustrated schematically in Figure 1C, contained RR120 an azo-dye that undergoes reductive bleaching (*E*_m_ = -0.4 V vs. SHE) to provide a readily quantifiable spectroscopic indicator of electron transfer into the proteoliposomes (Stikane et al., 2019).

**FIGURE 5:**
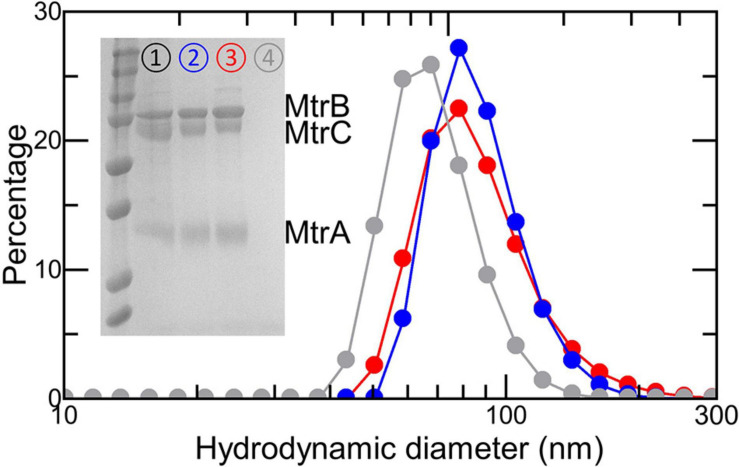
Characterization of (proteo-) liposomes. Size analysis by dynamic light scattering of RR120-containing liposomes with Ru-MtrC:MtrAB (blue), the MTR complex (red), or no proteins (gray). Proteoliposome concentrations estimated at 6 nM in 50 mM Tris:HCl and 10 mM KCl, pH 8.5. Inset shows Coomassie-stained SDS-PAGE gel loaded with the MTR complex (➀); RR120-containing proteoliposomes with the MTR complex (➁) or Ru-MtrC:MtrAB (➂); and RR120-containing liposomes (➃). Prestained protein ladder as in Figure 2C.

**FIGURE 6:**
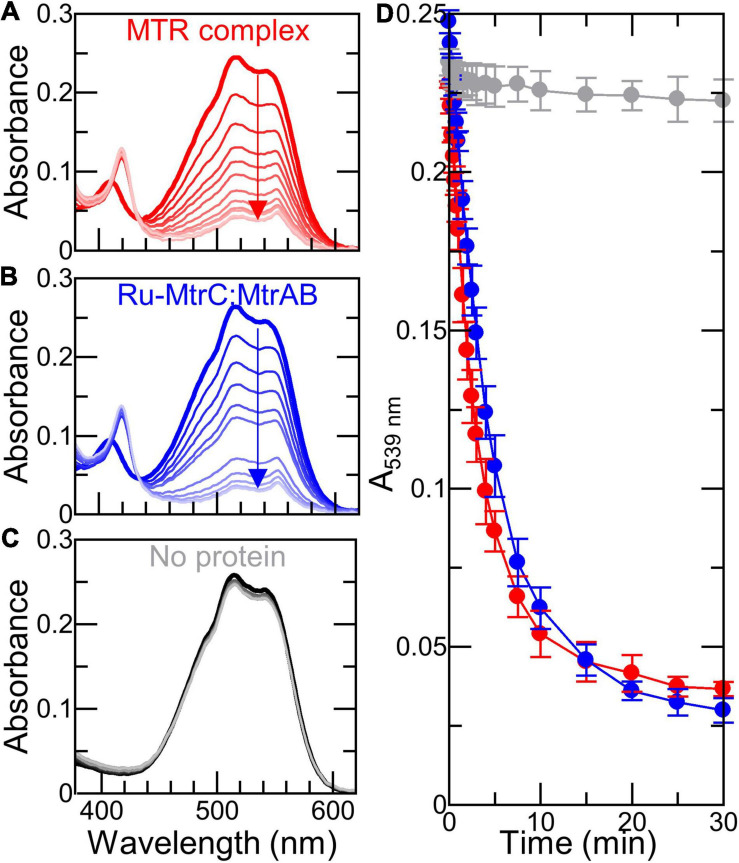
Dithionite-driven electron transfer across (proteo-)liposome bilayers **(A–C)** Spectra of 6 nM (proteo-) liposomes containing RR120 with indicated proteins before (thick line) and over 30 min following addition of 0.1 mM dithionite (thin lines); arrows indicate bleaching of RR120. Samples in 50 mM Tris:HCl and 10 mM KCl, pH 8.5. Scattering due to liposomes has been subtracted; see text for details. **(D)** Time course for bleaching of encapsulated RR120 from **(A–C)** with data points connected for clarity. Data are the average of *n* = 3 datasets and error bars show standard deviation.

Proteoliposomes loaded with RR120 were prepared as described in section “Materials and Methods.” SDS-PAGE confirmed that the proteins added during liposome formation were retained in the samples used to study electron transfer (Figure 5, inset). The presence of encapsulated RR120 was confirmed by a large peak from 460 to 570 nm in the absorbance spectra (Figures 6A,B) alongside a smaller Soret peak at 410 nm from Fe(III) heme. Deconvolution of these spectral features allowed the concentrations of dye and Mtr proteins to be calculated (Supplementary Figure 4 and Supplementary Table 2) and demonstrated that the ratio of complex to dye in both types of proteoliposome were similar (approximately 1:270). Dynamic light scattering revealed the size distributions of the proteoliposomes were independent of the incorporated protein (Figure 5). Zeta potential measurements gave values between -40 and -45 mV with no discernable dependence on the presence or absence of Mtr proteins. This finding is consistent with our estimate of < 5 complexes per liposome (Supplementary Table 2) with the complex having a footprint of approximately 40 nm^2^ in comparison with a proteoliposome surface area of approximately 30,000 nm^2^.

The 8 electrons necessary to reduce RR120 and its small extinction coefficient (ε_539 nm_ = 32.3 mM^–1^ cm^–1^) combined with the slow cumulative photoreduction rates for Ru-dye irradiation described above made it necessary for us to use different electron delivery systems with the (proteo-) liposomes. We chose the reductants for their previously described (Stikane et al., 2019) ability to produce rapid (<10 min) bleaching of internalized RR120 in MTR containing proteoliposomes. Addition of sodium dithionite to proteoliposomes containing Ru-MtrC:MtrAB or the MTR complex resulted in a rapid bleaching of the internalized RR120 (Figures 6A,B,D). In the absence of Mtr proteins, there was very little bleaching of the dye over the same time period (Figures 6C,D). Thus, Ru-MtrC:MtrAB is an effective conduit for electron transfer across the lipid bilayer. Furthermore, the rate of electron transfer through Ru-MtrC:MtrAB is comparable with that of the MTR complex (Figure 6D).

**FIGURE 7:**
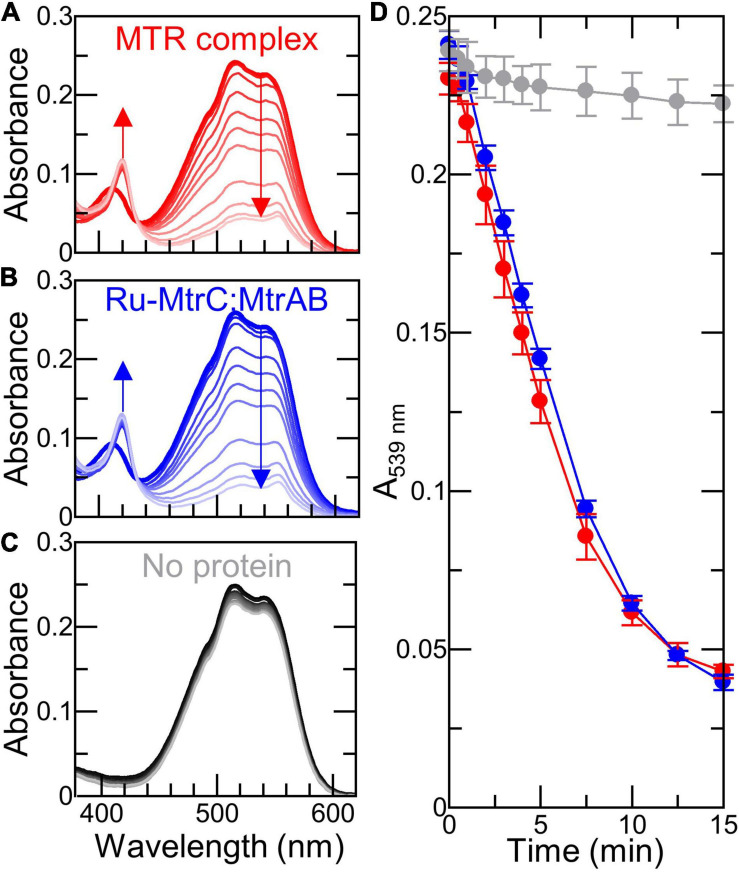
Light-driven electron transfer across (proteo-)liposome bilayers with graphitic N-doped carbon dots. **(A–C)** Spectra of 6 nM (proteo-) liposomes containing RR120 with indicated proteins before (thick line) and over 15 min irradiation (thin lines) with visible light (2.5 kW m^− 2^); arrows indicate reduction of hemes and bleaching of RR120. Samples in 50 mM Tris:HCl, 10 mM KCl, 25 mM EDTA, AND 10 μg ml^− 1^ graphitic N-doped carbon dots, pH 8.5. Scattering contributions from carbon dots and liposomes have been subtracted; see text for details. **(D)** Time course showing bleaching of encapsulated RR120 in (proteo-) liposomes from **(A–C)** with data points connected for clarity. Data are the average of *n* = 3 datasets, and error bars show standard deviation.

A final series of experiments explored the ability of graphitic N-doped carbon dots to support light-driven transmembrane electron transfer. These nanoparticles support rapid photoreduction when irradiated with white light (*λ* > 400 nm) in the presence of EDTA as sacrificial electron donor (Martindale et al., 2017). When our RR120 proteoliposomes containing Ru-MtrC:MtrAB or MTR complex were irradiated in the presence of EDTA, the rapid bleaching of RR120 was observed (Figures 7A,B). The complexes supported comparable rates of bleaching indicative of equivalent rates of electron transfer through Ru-MtrC:MtrAB and the MTR complex. Photoreduction of the Mtr hemes was also apparent through changes in the Soret band, specifically the red shift and increase of intensity. Almost all hemes appeared to be reduced in the first minute, indicating that electron transfer between (Ru-)MtrC and MtrAB is fast compared with the reduction of RR120. In the absence of Mtr proteins, liposomes containing RR120 showed very little evidence for dye bleaching over the same time period (Figure 7C). Thus, bleaching of the dye is dependent on electron transfer through the Mtr biomolecular wires.

Equivalent experiments performed in the absence of carbon dots or EDTA showed negligible reduction of heme and no bleaching of the encapsulated RR120 (Supplementary Figure 8). Furthermore, there was no detectable increase in the rate of RR120 bleaching in Ru-MtrC:MtrAB liposomes compared with MTR liposomes (Figure 7D). We conclude that Ru-MtrC makes very little contribution to photochemical electron production in these experiments and that carbon dots are the primary driver of the observed photochemistry.

By two methods of analysis, we find that transmembrane electron transfer by the Ru-MtrC:MtrAB complex is comparable with that by the MTR complex. This supports Ru-MtrC:MtrAB having a structure comparable with that of the MTR complex of *S. baltica* (Figure 1B), where MtrA Heme A10 and MtrC Heme C5 lie in close proximity to facilitate electron transfer across the interface between the two decaheme cytochromes. We note that the orientation(s) of the complexes in the proteoliposome membranes is not known. However, this is unlikely to affect our conclusions. Given the demonstrated similarity in structure of the MTR complex and Ru-MtrC:MtrAB, we expect that the populations of “inside-out” and “right-side-out” complex are similar for each type of proteoliposome. Both orientations are likely to occur as the protein complexes are present during formation of the liposome bilayers.

## Discussion

An increasing number and diversity of organisms are recognized to naturally transfer electrons between internal enzymes and external redox partners (Bose et al., 2014; White et al., 2016). The electron transfer pathways, evolved to allow survival in the absence of cell permeable electron acceptors, now inspire biotechnology to produce green energy and/or chemicals from material typically considered waste. Examples include the remediation of water-borne organic “waste” molecules coupled to electricity production in microbial fuel cells, and the microbial electrosynthesis of valued chemicals from CO_2_ and N_2_ driven by electricity from renewable sources (Chiranjeevi and Patil, 2020). These strategies rely on electron exchange between bacteria and electrodes. However, until recently, there has been very little molecular understanding of the proteins essential for electron exchange across bacterial outer membranes. It is now apparent that outer membrane-spanning complexes with a *c*-type cytochrome inside a beta-barrel porin protein play a major role in such electron transfer for numerous and phylogenetically diverse Gram-negative bacteria (Hartshorne et al., 2009; White et al., 2016; Edwards et al., 2020). Biochemical details are beginning to emerge for the porin-cytochrome fusion exemplified by CYC2 of *Acidithiobacillus ferroxidans* (Yarzabal et al., 2002; Castelle et al., 2008) the PioAB proteins of *Rhodopseudomonas palustris* TIE-1 (Gupta et al., 2019; Li et al., 2020) and the PCA complexes of *Geobacter sulfurreducens* (Liu et al., 2014). However, it is for *Shewanella* species that trans-outer membrane electron transfer is perhaps best described at the molecular level (e.g., Figures 1A,B). Notably, the MTR complex provides the primary, and bidirectional, route for electron transfer across the bacterial outer membrane (Ross et al., 2011; Shi et al., 2012).

Recent resolution of the molecular structure of an MTR complex (Edwards et al., 2020) paves the way for its rational engineering to facilitate electrical interfacing of *Shewanella*, or heterologous hosts, with external redox partners. The present study was informed by that structure. We have engineered MtrC for photoreduction by labeling the external surface with a Ru(II)-dye photosensitizer adjacent to the terminal Heme C10. Photo-energized electrons transfer from the dye to MtrC, and subsequently to MtrAB. Furthermore, the bespoke biomolecular wire created by mixing water-soluble Ru-MtrC with the lipid-soluble MtrAB porin:cytochrome complex is indistinguishable from purified MTR in both its structure and ability to transfer electrons across lipid bilayers. Our results can now inform the engineering of bacteria for inclusion in novel biohybrid materials with bespoke transmembrane biomolecular wires. Not only do they provide insight into the cellular pathway for MTR assembly, they suggest bespoke MTR complexes may be assembled in a modular fashion on the surface of live bacteria. We consider both topics below.

Precise details of the cellular MTR assembly pathway are lacking at the present time. In MR-1, the *mtrC*, *mtrB*, and *mtrA* genes encoding for the components of the MTR complex are co-transcribed. The translated peptides are transported in an unfolded state, through the Sec pathway, into the periplasm. The *Shewanella* cytochrome *c* maturation pathway folds MtrA and MtrC and attaches 10 hemes to each protein (Jin et al., 2013). MtrB is then combined with periplasmic MtrA to form the MtrAB complex through a process that may be initiated in the periplasm (Schicklberger et al., 2011). By contrast the type II secretion pathway secretes folded MtrC (Shi et al., 2008) to the external cell surface where it resides as a lipoprotein (Myers and Myers, 2001). The MTR complex forms when the lipoprotein MtrC, anchored to the external cell surface, associates with MtrAB in the outer membrane. By demonstrating that soluble MtrC, lacking the N-terminal lipid attachment site (Lockwood et al., 2018) associates with MtrAB to form a complex indistinguishable from MTR, this study reveals that the interfacial contacts between MtrC and MtrAB are sufficient to define and stabilize a functional complex. The lipid anchor carried by genomically encoded MtrC appears to have no structural role. That anchor may ensure that secreted MtrC, synthesized in a process requiring significant investment of cellular resources, diffuses only across the cell surface, essentially in 2D, until it encounters and associates with the MtrAB complex to then perform its role in anaerobic respiration.

**FIGURE 8:**
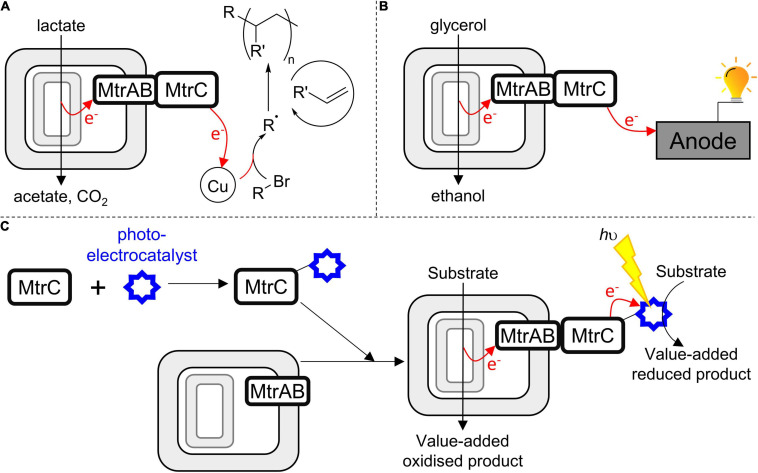
Harnessing the MTR complex to couple internal (biological) and external (synthetic) catalysts in a closed redox cycle. **(A)** *S. oneidensis* as a living electrode driving an exogenous copper-based catalyst to produce radicals, these initiate olefin polymerization (Fan et al., 2018). **(B)** *S. oneidensis* performing electrode-assisted glycerol oxidation (Flynn et al., 2010). **(C)** A strategy to engineer bacteria for semiartificial photosynthesis motivated by this work; soluble MtrC is functionalized with a photoelectrocatalyst and then assembles spontaneously with bacteria presenting MtrAB in their outer membrane; allowing intracellular substrate oxidation to be coupled to extracellular photoelectrocatalysis.

With regard to developing novel biotechnology for greener chemical synthesis, much inspiration is provided by bacteria, such as *S. oneidensis* MR-1, which naturally couple internal and external redox processes. Electrons, for example, derived from lactate oxidation by *S. oneidensis* MR-1 (Figure 1A) have been delivered (Fan et al., 2018) to an exogenous extracellular catalyst for atom-transfer radical polymerization (Figure 8A). Separately, genetic engineering of *S. oneidensis* introduced new metabolism and demonstrated the production of value-added substances more oxidized than the substrates (Figure 8B) when glycerol was converted to ethanol (Flynn et al., 2010) and acetoin produced from glucose (Bursac et al., 2017). Both studies benefited from electrode-assisted fermentation with the liberated electrons passed to anodes. In the future, we envisage value-adding half-reactions inside the bacteria will be coupled to value-adding half-reactions that occur outside the cells such that the concepts above are integrated in a single biotechnology. In such a scenario, the performance may be enhanced if MtrC is functionalized with an external electrocatalyst to facilitate electron exchange with the cells. Furthermore, using external photoelectrocatalysts will have the additional advantage of allowing reactions to be driven by the energy of sunlight to achieve semiartificial photosynthesis in a living organism (Figure 8C).

While such opportunities are exciting, the direct labeling of bacteria with (photo-)electrocatalysts is likely to provide a challenge, not least the possibility that labeling reactions might compromise cell viability. By demonstrating spontaneous formation of a stable and functional complex able to transfer electrons across a lipid bilayer, this work suggests an alternative route to assembling such biohybrids (Figure 8C). In the first step, selective *in vitro* functionalization of purified, soluble MtrC would be carried out under optimal conditions. Then, spontaneous association of functionalized MtrC with cells presenting MtrAB in their outer membrane would afford assembly of the desired biohybrid materials through a strategy that makes a virtue of the intrinsic modularity of the MTR complex. With regard to this strategy, do conditions exist whereby functional MtrC:MtrAB complexes can be assembled by combining soluble, functionalized MtrC with bacteria presenting MtrAB in their outer membranes? Experiments to assess this possibility are ongoing in our laboratories. We also aim to engineer the coupling of MtrC to photosensitizers more sustainable than Ru(II)-dyes, and that support faster light-driven electron accumulation in the protein.

In conclusion, we have demonstrated photoreduction of MtrC photosensitized by covalent linkage to an inorganic dye. We have also established that the structure and electron transfer properties of the complex formed when functionalized MtrC associates with MtrAB *in vitro* are comparable with those of the native MTR complex. Together, these observations lay the foundations for rational engineering of the MtrC:MtrAB complex for novel synthetic biology for enhanced biotechnology of *Shewanella* and heterologous hosts such as *Escherichia coli* (Schuergers et al., 2017; Su and Ajo-Franklin, 2019).

## Data Availability Statement

The datasets presented in this study can be found in online repositories. The names of the repository/repositories and accession number(s) can be found below: http://www.sasbdb.org/, SASDL97, SASDLA7; https://research-portal.uea.ac.uk/en/persons/julea-butt, no accession number.

## Author Contributions

JB, TC, SP, LJ, and ER designed research. SP, ME, JW, and AM performed the research. CC provided carbon dots. SP, JW, ME, AM, and TC analyzed data. JB, SP, ME, and TC wrote the manuscript. All authors provided critical feedback on the results and manuscript.

## Conflict of Interest

The authors declare that the research was conducted in the absence of any commercial or financial relationships that could be construed as a potential conflict of interest.

## Publisher’s Note

All claims expressed in this article are solely those of the authors and do not necessarily represent those of their affiliated organizations, or those of the publisher, the editors and the reviewers. Any product that may be evaluated in this article, or claim that may be made by its manufacturer, is not guaranteed or endorsed by the publisher.
