# Supplementary material for: Bespoke Biomolecular Wires for Transmembrane Electron Transfer: Spontaneous Assembly of a Functionalized Multiheme Electron Conduit
Source: Front Microbiol. 2021 Aug 16;12:714508. doi: 10.3389/fmicb.2021.714508 (PMC8415449; doi:10.3389/fmicb.2021.714508)
Supplement: Supplementary file 1 [file Presentation_1.pdf]

Supplementary Material

1 Estimating Liposome Concentration

The liposomes' size, determined by DLS, and other known properties of the lipids used were used to estimate the concentration of liposomes in the samples. Details of the polar lipid extract provided by Avanti (Table S1) were used to calculate an average molecular weight for lipid in the extract.

Table S1. Composition of Polar Lipid Extract (Avanti).

Component	Percentage by weight	Average molecular weight (Da)	Percentage by number
Phosphatidylethanolamines (PE)	67	719.3	71.3
Phosphatidylglycerols (PG)	23.2	761.1	23.4
Cardiolipin (CA)	9.8	1430.0	5.3

$$\frac{(71.3\times 719.3\text{ Da})+(23.4\times 761.1\text{ Da})+(5.3\times 1430.0\text{ Da})}{100} = 766.7\text{ Da}$$

Dynamic Light Scattering of the liposomes revealed size distributions with a mean diameter of approx. 100 nm, Fig. 5 main text. The width of a phospholipid bilayer[1] is approx. 4 nm, meaning the inner leaflets of these liposomes have diameters of approx. 92 nm. Using this diameter and the formula for the surface area of a sphere ($4\pi r^2$) the total surface area of a liposome can be estimated:

$$4\pi(50\text{ nm})^2 + 4\pi(46\text{ nm})^2 \approx 58000\text{ nm}^2$$

The number of lipids in one liposome can then be estimated from the average footprint[1] of a phospholipid is approx. 0.7 nm^2.

$$\frac{58000\text{ nm}^2}{0.7\text{ nm}^2} = 82866$$

Therefore each liposome has an approximate M_W of:

$$82866 \times 766.7\text{ Da} = 64\text{ MDa}$$

This M_w can be used calculate that the total number of liposomes that can be prepared from the 20 mg Polar Lipid Extract used in our studies.

$$\frac{2\times 10^{-2}\text{ g}}{6.4\times 10^{7}\text{ g mol}^{-1}} \approx 300\text{ pMol} = 1.8\times 10^{14}\text{ liposomes}$$

Therefore, after final resuspension of the liposomes in 1 mL their concentration is estimated as 300 nM.

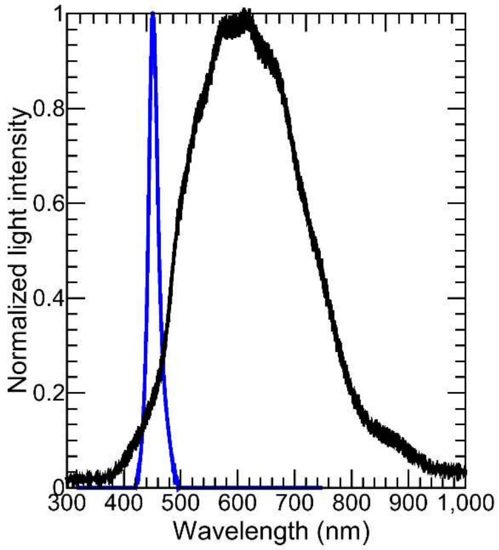

Supplementary Figure 1. Spectral distributions of light sources used in this work. Blue LED spectrum (blue) obtained from manufacturer, Krüss Cold Light source spectrum (black) measured by Dr Sam Rowe using a HR2000CG-UV-NIR Ocean Optics fibre optic spectrometer. Light intensity has been normalized for comparison.

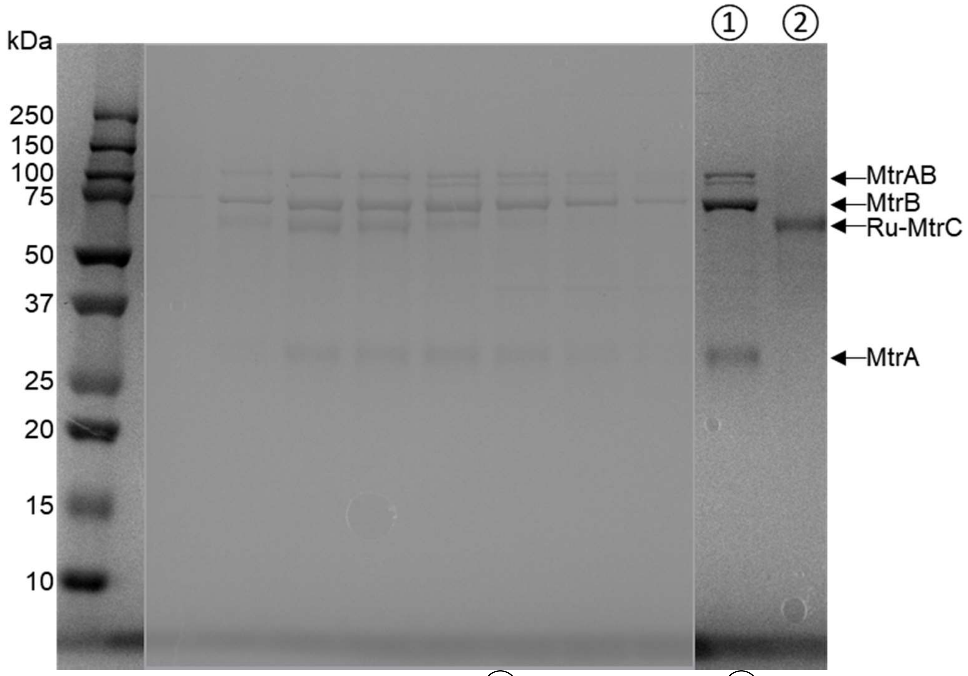

Supplementary Figure 2. SDS-PAGE of MtrAB (①) and Ru-MtrC (②). Gel has been Coomassie stained. Lanes containing samples not relevant to this work are partially concealed.

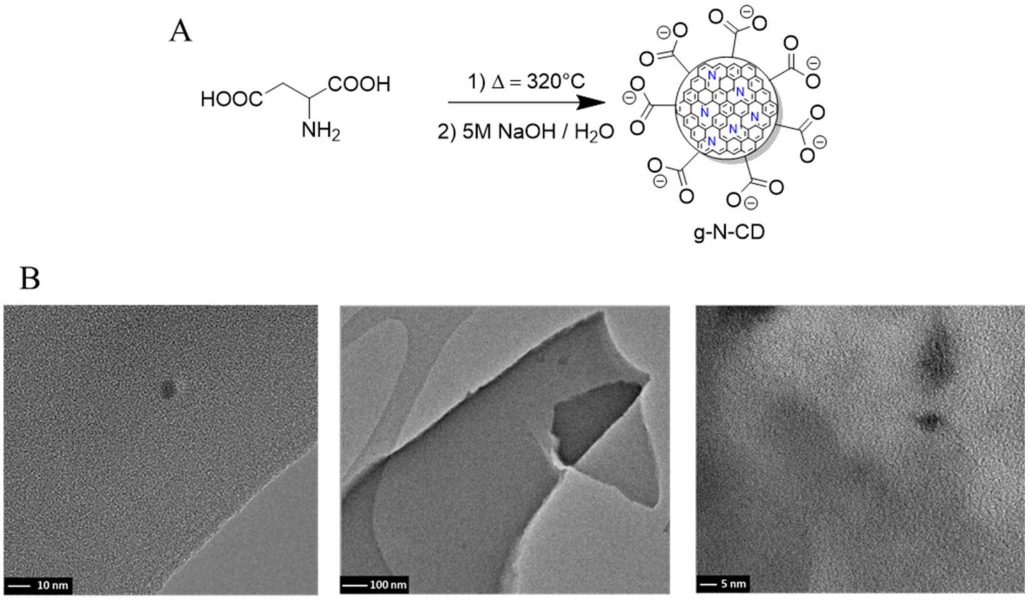

Supplementary Figure 3. Description of graphitic N-doped Carbon Dots. (A) Synthesis of Carbon Dots by pyrolysis of aspartic acid[2]. (B) Transmission electron microscopy images of Carbon Dots. Images were collected on a Thermo Scientific (FEI) Talos F200X G2 TEM machine at an accelerating voltage of 200 kV. These images show the Carbon Dots to have a diameter of 3.1 $\pm$ 1.1 nm consistent with those reported by Martindale et al [2]. A molecular weight of 21 kDa is calculated based on density of graphite (2.266 g cm^{-3}).

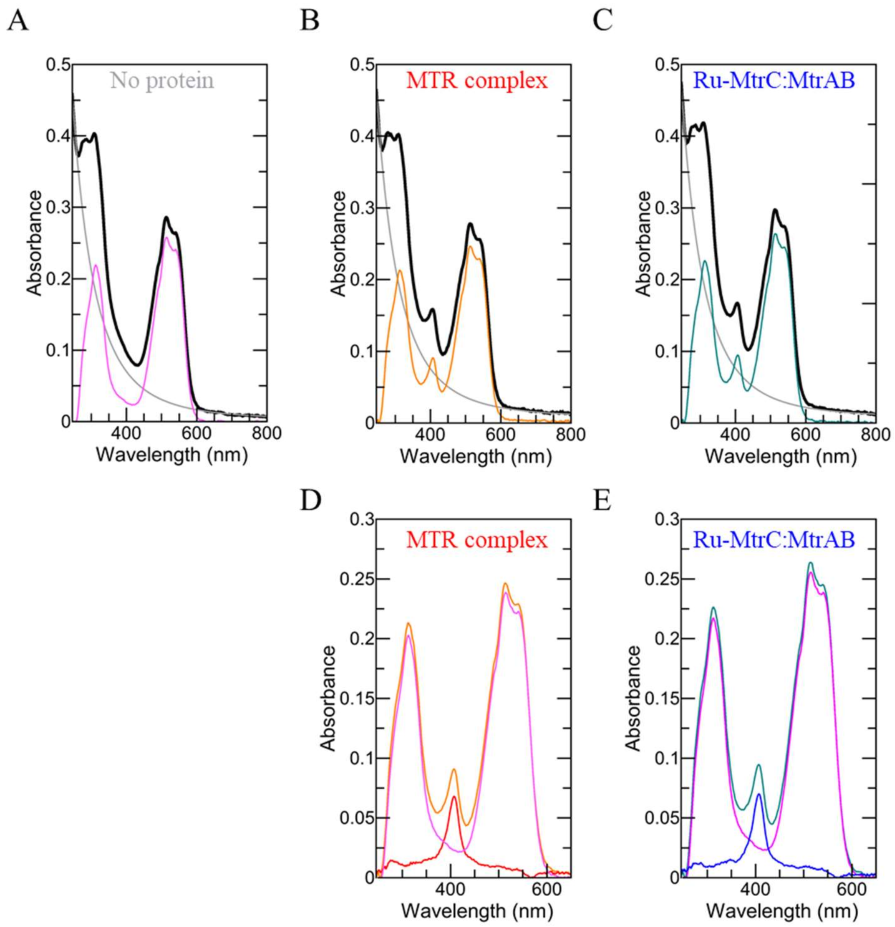

Supplementary Figure 4. Deconvolution of liposome spectra. (A-C) Spectra of 6 nM liposomes with encapsulated RR120 and the indicated proteins (black) and simulated Rayleigh scattering from the liposomes (gray), see text for details. The absorbance due to RR120 and the Mtr proteins (pink/orange/teal) was obtained by subtraction of the scattering contribution from the measured spectrum. (D-E) Total absorbance (orange/teal) of RR120 and Mtr proteins as indicated (data from panels B and C), contributions from RR120 (pink) and the indicated Mtr complex (red/blue) allow the concentrations of each species to be quantified (Table S2).

Table S2. Composition of the liposome systems of Supplementary Figure 4.

Protein Incorporated	[RR120] (μM)	[Mtr complex] (nM)	Mtr:RR120 ratio	Mtr per liposome
MTR Complex	6.8	25.6	1:266	4.3
Ru-MtrC:MtrAB	7.3	26.3	1:278	4.4
No protein	7.4	-	-	-

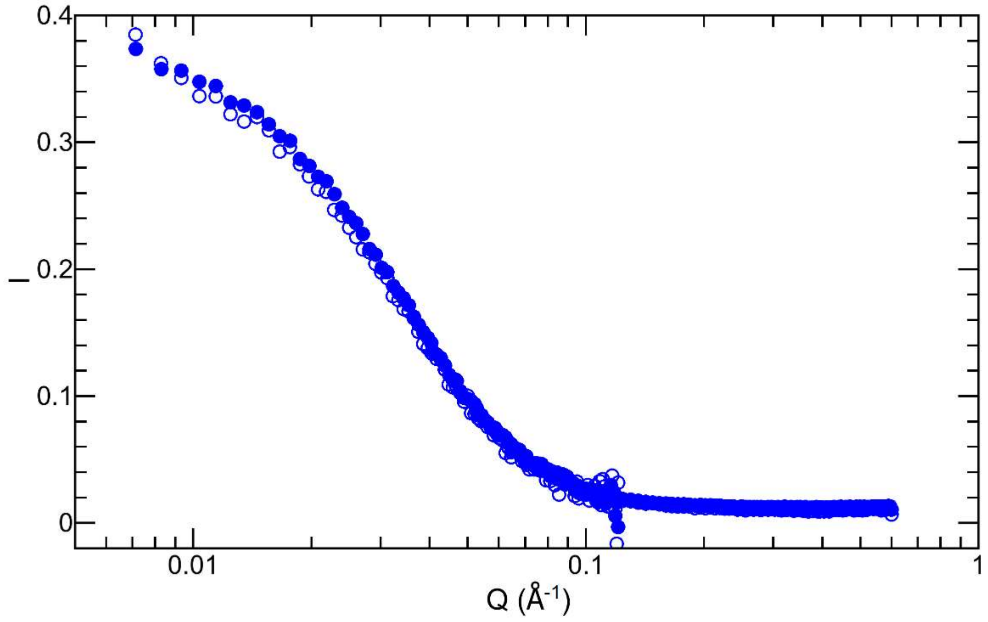

Supplementary Figure 5. Buffer-subtracted scattering curves for Ru-MtrC:MtrAB at 6.3 mg/mL (filled circles) and 3.1 mg/mL after scaling up by a factor of 2.03 (open circles).

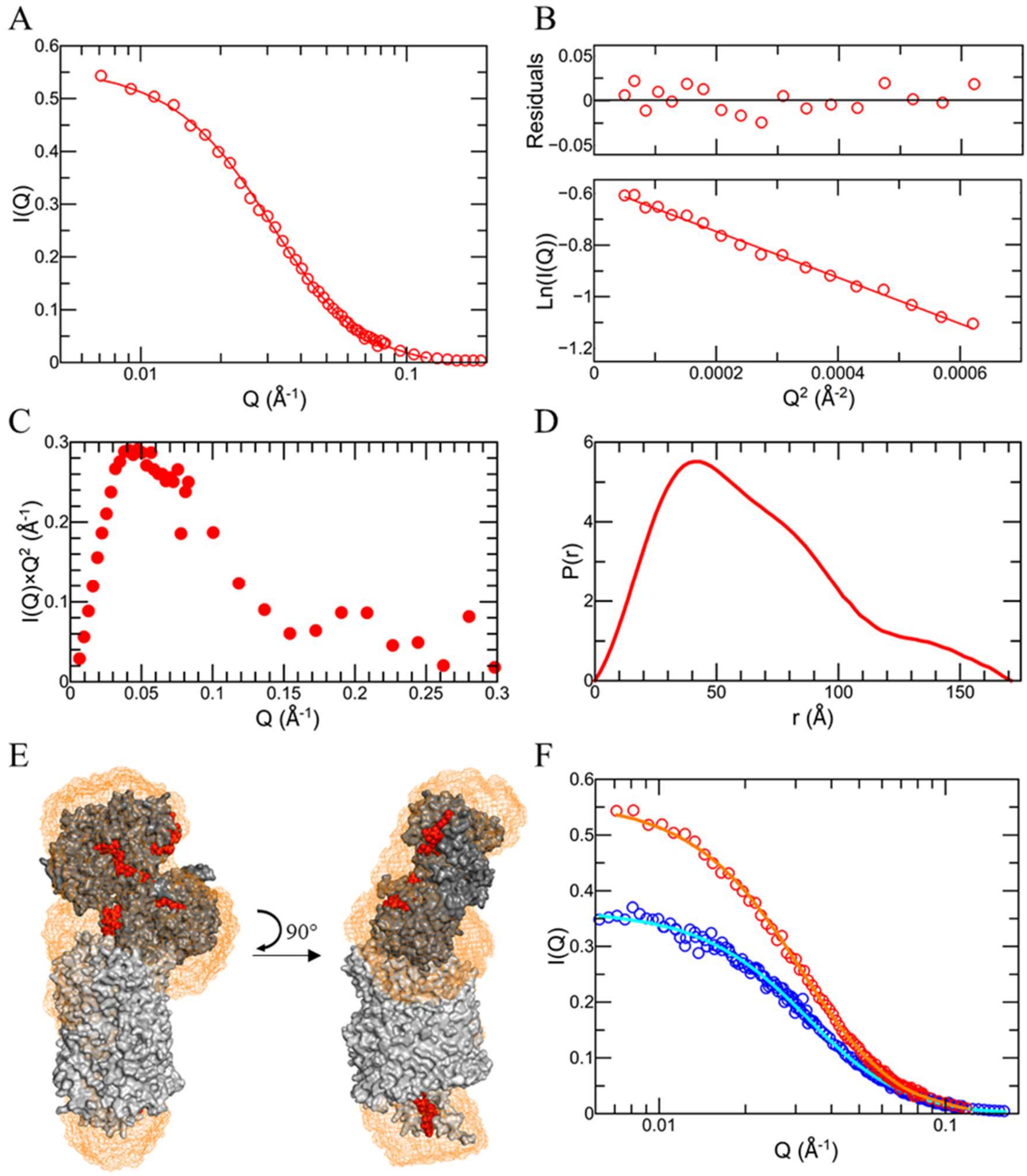

Supplementary Figure 6. SANS analysis of the MTR complex. (A) Scattering data for the MTR complex shown as circles with the fit to the P(r) curve shown as a line. (B) Guinier region of scattering curve, lower panel shows data as circles and a linear fit as a line. Upper panel shows residuals from fitting. (C) Kratky plot. (D) P(r) curve produced for the MTR complex from data reported previously in Edwards *et al.*[3]. (E) Molecular envelope of the MTR complex (orange mesh) generated by DAMMIN and aligned with the homology model of the MTR complex from *S. oneidensis* MR-1 (gray with red hemes). (F) Scattering data for the MTR complex (red circles) and Ru-MtrC:MtrAB (blue circles, Fig. 3A main text). Theoretical scattering curve for the molecular envelope of the MTR complex as presented in (E) (orange line) and Ru-MtrC:MtrAB as presented in Fig. 3E main text (cyan line).

Tables S3. SANS sample, data collection, and processing parameters.

**S3A Sample details**	Ru-MtrC:MtrAB	MTR complex
Organism	*Shewanella oneidensis* MR-1	*Shewanella oneidensis* MR-1
UniProt sequence ID (residues in construct)	MtrC - Q8EG34 (22-671) (Y657C), MtrA - Q8EG35 (35-333), MtrB - Q8CVD4 (22-697)	MtrC - Q8EG34 (22-671), MtrA - Q8EG35 (35-333), MtrB - Q8CVD4 (22-697)
Extinction coefficient at 410 nm (M^{-1} cm^{-1})	2627000	2627000
$\bar{v}$ from chemical composition (cm^3 g^{-1}) (not including hemes)	0.718	0.718
Particle contrast from sequence and solvent constituents, $\Delta\bar{\rho}$ ($\rho_{protein}$ - $\rho_{solvent}$; 10^{10} cm^{-2})	1.79	1.79
Molecular weight from chemical composition (Da)	190885	189282
Average concentration in combined data frames (mg ml^{-1})	6.3	9.3
Solvent (solvent blanks taken from dialysis buffer)	20 mM HEPES, 100 mM NaCl, 2.8 mM Fos-choline 12, 13% D_2O, pH 7.8	

**S3B SANS data-collection parameters**	Ru-MtrC:MtrAB	MTR complex
Instrument/data processing	Institut Laue-Langevin D22 beamline	Institut Laue-Langevin D22 beamline
Wavelength (Å)	6 ±10%	6 ± 10%
Beam size (mm)	10 mm × 7 mm	10 mm × 7 mm
Collimation; sample-detector length (m)	(17.6; 17.6), (8; 8) (2.8; 1.4)	(8; 8), (2.8; 1.4)
q measurement range ($Å^{-1}$)	0.003 - 0.6	0.007 - 0.55
Absolute scaling method	Direct (flux at sample position)	Direct (flux at sample position)
Normalization	Upstream monitor count	Upstream monitor count
Exposure time	60 s to 2 h depending on sample concentration, contrast and instrument configuration	60 s to 1 h depending on sample concentration, contrast and instrument configuration
Sample configuration	1 mm pathlength Suprasil Quartz rectangular cuvettes	1 mm pathlength Suprasil Quartz rectangular cuvettes
Sample temperature (°C)	10	10

**S3C Software employed for data reduction, analysis and interpretation**	Ru-MtrC:MtrAB	MTR complex
SANS data reduction	Grasp (C. Dewhurst), SANS reduction Igor Macros (S. Kline) and correction as below.	
Constant added to correct buffer oversubtraction (cm^{-1})	0.0013	0.0032
Extinction coefficient estimate	Determined by pyridine hemochromagen assay[4]	
Calculation of $\Delta\bar{\rho}$ and $\bar{v}$ values	Ultrascan II utilities	
Basic analyses: Guinier, P(r)	Primus from ATSAS 3.0.1[5]	
Shape/bead modelling	DAMAVER[6], DAMMIN[7] and SUPCOMB[8]	
Atomic structure modelling	N/A	
Missing sequence modelling	N/A	
Three-dimensional graphic model representations	PyMOL 2.3.4 Win10	

**S3D Structural parameters**	Ru-MtrC:MtrAB	MTR complex
Guinier analysis		
I(0) (cm^{-1})	0.36 $\pm$ 0.0026	0.56 $\pm$ 0.0035
R_g (Å)	46.9 $\pm$ 0.58	51.6 $\pm$ 0.53
Q (min) (Å^{-1})	0.00621	0.00715
QR_g max	1.29	1.29
Coefficient of correlation R^2	0.966	0.993
P(r) analysis		
I(0) (cm^{-1})	0.3654	0.5597
R_g (Å)	48.2	51.5
D_{max} (Å)	166	170.6
Q range (Å^{-1})	0.0062 - 0.161	0.0071 - 0.1191
χ^2 (total estimate from GNOM)	0.7641	0.7452
M from I(0) (ratio to predicted)	208328 (1.09)	219528 (1.16)
Porod volume (Å^{-3}) (ratio V_r/calculated M)	153711	234444

**S3E Shape model-fitting results**	Ru-MtrC:MtrAB	MTR complex
**DAMMIF** (default parameters, 20 calculations)		
q range for fitting (Å^{-1})	0.0062 - 0.161	0.0071 - 0.1191
symmetry, anisotropy assumptions	P1, none	P1, none
χ^2 range	2.761 - 2.832	1.991 - 2.025
**DAMSEL**		
Ensemble resolution (from SASRES) (Å) [9]	52 ± 4	53 ± 4
NSD (standard deviation)	1.106 (0.08)	1.041 (0.213)
Number of models discarded	1 (out of 20)	2 (out of 20)
**DAMSUP**		
NSD range	0.814 - 1.135	0.524 - 1.315
**DAMMIN**		
q range for fitting (Å^{-1})	0.0062 - 0.161	0.0071 - 0.1191
Symmetry, anisotropy assumptions	P1, none	P1, none
χ^2 from DAMMIN (derived using χ^2 formula)	2.748 (0.0344)	1.972 (0.0225)
**SUPCOMB**		
NSD	2.05	2.72

**S3F SASBDB IDs**	Ru-MtrC:MtrAB	MTR complex
	SASDL97	SASDLA7

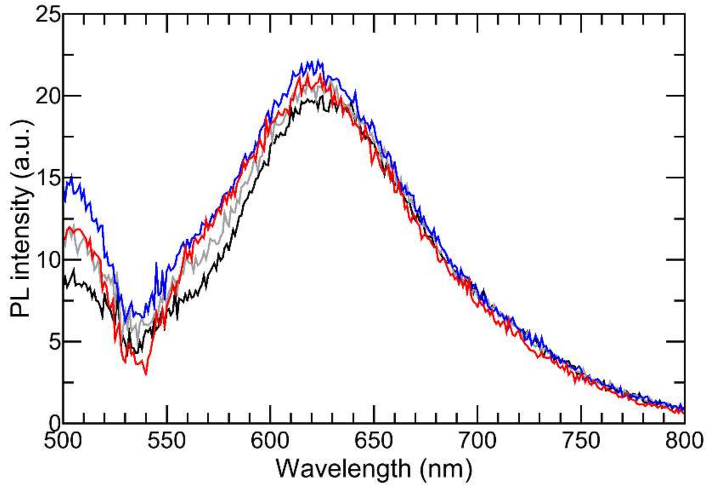

Supplementary Figure 7. Photoluminescence emission spectra for 1 μM Ru-MtrC before (black) and after addition of MtrAB to 0.5 μM (gray), 1 μM (blue) or 2 μM (red). Samples measured in sealed 1 mL quartz fluorescence cuvettes in anaerobic 50 mM sodium phosphate, 50 mM NaCl, 5 mM LDAO, pH 7.5.

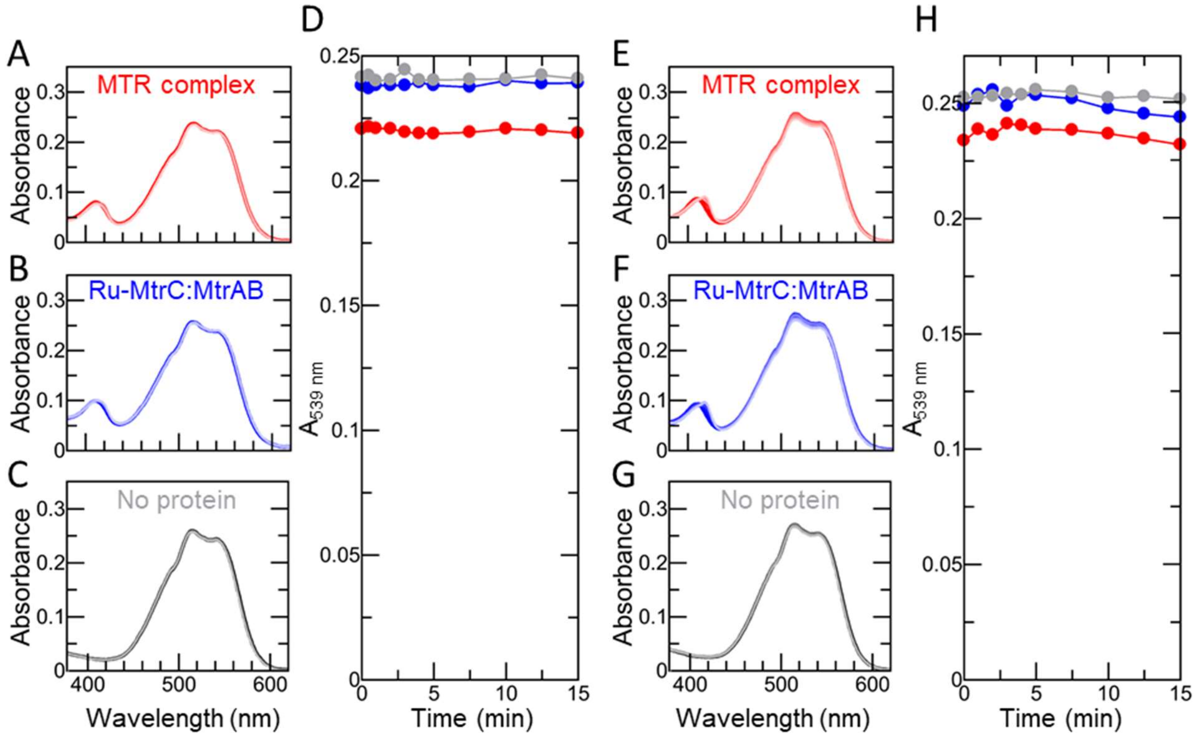

Supplementary Figure 8. Irradiation of 6 nM (proteo-)liposomes containing RR120 in the absence of graphitic N-doped Carbon Dots (A-D) or in the absence of EDTA (E-H). (A-C) Spectra as indicated of RR120-containing (proteo-)liposomes before (thick line) and during 15 min irradiation (thin lines) with visible light (2.5 kW m^{-2}) in 25 mM EDTA, 50 mM Tris:HCl, 10 mM KCl, pH 8.5. (D) Absorbance (539 nm) time course due to RR120, data from panels A-C. (E-G) Spectra as indicated of RR120-containing (proteo-)liposomes before (thick line) and during 15 min irradiation (thin lines) with visible light (2.5 kW m^{-2}) in 10 µg/mL Carbon Dots, 50 mM Tris:HCl, 10 mM KCl, pH 8.5. (H) Absorbance (539 nm) time course due to RR120, data from panels E-G. Scattering contributions from liposomes and Carbon Dots (where present) have been subtracted as described in main text.

References

1. Nagle, J.F. and Tristram-Nagle, S., (2000). Structure of lipid bilayers. Biochim. Biophys. Acta 1469, 159-195.

2. Martindale, B.C.M., Hutton, G.A.M., Caputo, C.A., Prantl, S., Godin, R., Durrant, J.R. and Reisner, E., (2017). Enhancing light absorption and charge transfer efficiency in carbon dots through graphitization and core nitrogen doping. Angew. Chem. 56, 6459-6463.

3. Edwards, M.J., White, G.F., Butt, J.N., Richardson, D.J. and Clarke, T.A., (2020). The crystal structure of a biological insulated transmembrane molecular wire. Cell 181, 665-673.

4. Barr, I. and Guo, F., (2015). Pyridine hemochromagen assay for determining the concentration of heme in purified protein solutions. Bio Protoc. 5, e1594.

5. Konarev, P.V., Volkov, V.V., Sokolova, A.V., Koch, M.H.J. and Svergun, D.I., (2003). PRIMUS: a Windows PC-based system for small-angle scattering data analysis. J. Appl. Crystallogr. 36, 1277-1282.

6. Volkov, V.V. and Svergun, D.I., (2003). Uniqueness of *ab initio* shape determination in small-angle scattering. J. Appl. Crystallogr. 36, 860-864.

7. Svergun, D.I., (1999). Restoring low resolution structure of biological macromolecules from solution scattering using simulated annealing. Biophys. J. 76, 2879-2886.

8. Kozin, M.B. and Svergun, D.I., (2001). Automated matching of high- and low-resolution structural models. J. Appl. Crystallogr. 34, 33-41.

9. Tuukkanen, A.T., Kleywegt, G.J. and Svergun, D.I., (2016). Resolution of ab initio shapes determined from small-angle scattering. IUCrJ 3, 440-447.
